# Lectures on the Diagnosis and Treatment of Diseases of the Lungs

**Published:** 1841-07-03

**Authors:** W. W. Gerhard


// MEDICAL EXAMINER.

DEVOTED TO MEDICINE, SURGERY, AND THE COLLATERAL SCIENCES.

No. 27.] PHILADELPHIA, SATURDAY, JULY 3, 1841. [Vol. IV.

### LECTURES ON THE DIAGNOSIS AND TREATMENT OF DISEASES OF THE LUNGS.

BY W. W. GERHARD, M. D.

### LECTURE XV.

#### PULMONARY HÆMORRHAGE.

Hæmorrhage from the lungs, in most instances, consists merely in an exudation of blood from the bronchial membrane, and is symptomatic of deeper seated diseases of the lungs; but as it arises from several causes, and may depend upon simple excitement of the circulation, or disease of the heart, as well as upon positive disease of the lungs, it requires, on some accounts, a separate examination. The connection of hæmoptysis with different stages of tuberculous disease has been already explained in the lectures upon phthisis; the object of the present remarks is, therefore, hæmoptysis itself, considered as a separate disorder, and not a mere symptom of other and deep seated pulmonary affections.

It may be divided into the hæmorrhage which is purely external, in which the blood comes directly from the mucous membrane, and is discharged externally; and into another variety, in which a portion of the blood escapes into the cellular tissue of the lungs, and forms little nuclei, which are of a deep red colour, and of almost a uniform appearance. These nuclei constitute the disease known under the name of pulmonary apoplexy, which is nothing more nor less than hæmorrhage from the vessels of the smaller bronchial tubes and vesicles, into the cellular tissue of the lung. It is, in general, attended with a flow of blood externally; but in some cases the effusion is strictly internal, and the disease is then indicated only by the dyspnœa and obstruction to the circulation.

There is some difference as to the causes of the slighter varieties of hæmoptysis and pulmonary apoplexy. The latter follows, in most cases, the sudden and violent congestions of the lung, which a disease of the heart or aorta naturally produces; or it arises from some other equally decided obstruction to the circulation; but the hæmorrhage which finds its way entirely to the surface, depends, in most cases, upon a less severe, but more persistent cause of irritation, seated in the lungs themselves.

In either variety of hæmorrhage there is, therefore, something more than a mere flow of blood to the head; there is a cause, either general or local, or both united, which determines the raptus toward the lungs, and then a discharge into the cellular tissue, and upon the surface of the bronchial membrane, or upon the latter alone. The first stage in the morbid chain is the congestion which may occur without the effusion of blood, or with hæmorrhage, into the cellular tissue, but not externally. Hence, the discharge of blood is in itself of no importance, except in the rare cases in which it is so considerable as to enfeeble the patient very much; the real mischief is the effect produced upon the pulmonary tissue. If there be an apoplectic extravasation, the mischief is more considerable than from simple congestion, and the secondary irritation much greater than when there is simply an arterial congestion, giving to the lung a bright vermillion-red colour. The secondary irritation may be merely a moderate inflammatory action in the part, or a tuberculous deposit; should the latter exist before the hæmorrhage, the lesion of the lung is simply a favouring cause, which increases the number, and favours the growth and softening of tubercles.

The symptoms and mode of attack of pulmonary hæmorrhage may begin in several ways. A patient may be either using strong and even violent exercise, which determines a sudden rush of blood towards the lungs, and the hæmorrhage then ensues,—or it may occur while the patient is perfectly quiet. Either of these modes of occurrence may coincide with tuberculous disease, but the latter is more frequently connected with it than the former. There is no difference in the symptoms of the hæmorrhage connected with tubercles or tuberculous diathesis, and that dependant upon other causes. The cases vary only according to their severity, and the previous health of the patient.

The general symptoms are perfectly the same as those of other hæmorrhages; hence they require but little special attention in a lecture devoted to pectoral disease. The heart is throbbing and quick, its contraction is accompanied, in many instances, with a bellows sound, which is extremely loud and strong. As the hæmorrhage is almost always of an active character, the face is unduly flushed, and the capillary circulation excited. These symptoms gradually decline after the flow of blood, except the action of the heart and arteries, which remains for a considerable time in its former state.

The local or pectoral symptoms are more immediately connected with our subject. If the hæmorrhage be slight, the patient complains only of a slight sense of tickling at the upper part of the trachea and the large bronchial tubes. If the hæmorrhage be considerable, the tickling is more constant and severe, and a sense of oppression is felt across the sternum, which seems to prevent the full expansion of the chest. As the hæmorrhage generally lasts for some time before it finally ceases, the tickling sensation continues, and even after the flow of fresh blood has completely ceased, the coagula continue to be expectorated, and keep up the same sensation of tickling, with the short irritated cough which naturally results from it. There is no pain from the hæmorrhage proper; the pain, if it exist, depends only on the accidental inflammation which sometimes follows the hæmorrhage; for the effused blood left in the cellular tissue of the lung may prove an irritating cause, like other foreign bodies.

The signs of auscultation are merely a loose sub-crepitant rhonchus, heard not only at the seat of the hæmorrhage, but throughout the bronchial tubes which contain blood. The bubbles are even looser and of thinner liquid than those formed by mucus. The percussion is rendered slightly dull if there be a large apoplectic extravasion, or much congestion of the surrounding tissue. The evidence of hæmoptysis does not rest, however, upon physical signs, but on the external discharge of blood; and in those rare cases in which the blood is extravasated into the cellular tissue of the lungs without appearing externally, no certain conclusions can be drawn from auscultation.

The course of hæmorrhage is rarely towards a fatal termination, unless the first gush of blood should prove fatal: even this is not common, except in advanced phthisis, when the hæmorrhage comes from a large vessel crossing a cavity, and is not the result of exudation from smaller vessels and the finer bronchial tubes. This accident is then strictly dependant upon phthisis.

The treatment of hæmorrhage from the lungs does not differ materially from analogous affections, excepting that it is connected with other diseases of the lungs, especially pulmonary phthisis, in which case the treatment is a mere appendage to that of this disease. There is generally little difficulty in suppressing the bleeding; but after this has been brought about, our object is to prevent its return, and check the subsequent fever, which is not only attended with some danger in itself, but is a favouring cause of tubercles.

### PERICARDITIS.

The frequency of pericarditis has been known only of late years. It was formerly supposed that it was a very rare disease, and attended with symptoms of great severity, terminating in most instances fatally. The late investigations have, however, proved that such is not the case, but that pericarditis is an extremely frequent disorder, not much more severe in many instances than pleurisy, and very often not recognisable by any rational symptoms. The most important of these researches were those of Dr. Louis, published in the year 1826. He then thought that the only conclusive evidence of previous pericarditis was adhesions between the two surfaces of the serous membrane, but it is now ascertained that in most instances of slight pericarditis, there is merely a deposit of white opaque lymph in patches upon the surface of the heart, and not an actual adhesion; the observations of Dr. Lous did not, therefore, in all probability, include more than a very small proportion of cured cases of the disease.

At first, the only evidence that pericarditis was curable, depended upon the traces left behind it, and discovered in the bodies of patients dead of other diseases; but as the symptoms of pericarditis became better known, it was recognised in most cases during life, and could be traced throughout its whole course, unto complete recovery. As before attention was directed to the subject, it was believed that it was always, or nearly always, attended with severe and dangerous symptoms; it is now known that, in the great majority of cases, the symptoms are but slight, and that not unfrequently it cannot be recognised; or, in other words, it is latent, except through the aid of the physical signs. There are even some slight cases in which the latter are by no means conclusive, so that very few disorders are as often overlooked as pericarditis. It is, therefore, the more necessary to pay close attention to those signs which can be detected, otherwise the slighter forms of the disease may pass almost insensibly into the more severe and dangerous varieties.

The *anatomical lesions* of pericarditis are similar to those of other serous membranes, with slightdifferences depending upon the peculiar structure and situation of the membrane. At first the natural serous secretion is but little altered, and is even less abundant than usual; almost at the same time, or soon after, a slight formation of lymph takes place, at first in the form of little points or dots scattered over the surface of the membrane, which gradually become more and more numerous, until they unite in one uniform membrane, and cover the whole surface of the pericardium. The lymph is in this stage soft, not much thicker than wrapping paper; when it becomes more abundant, certain portions sink in the form of shreds to the lower part of the liquid, and the coating which remains attached to the heart becomes roughened, and assumes a honey-combed appearance, which depends upon the continual motion of the heart, and the drawing asunder of the two coats of false membrane. After the process of cure commences, the serum is first absorbed as in other cases of inflammation of serous membranes, and the false membranes become consolidated into new formed tissue. This assumes the appearance of ordinary serous membrane when it forms partial adhesions between these two surfaces of the membranes; of cellular tissue when the adhesion is so extensive as to block up the whole or a large part of the cavity, and prevents the passage of serum between the two opposing portions of the pericardium; of opaque white patches of firmer tissue, sometimes semi-cartilaginous which form a close adhesion to the membrane, but may still be removed from it by strongly scraping with a knife: or lastly, of a simple opacity of the membrane depending not upon a deposit on its surface, but on the effusion of new matter in its substance, or on its adherent surface.

The process of absorption, after the more acute periods of the inflammation are passed, is generally more slow than that of effusion; but if there is little lymph, and a large proportion of serum, it sometimes takes place with great rapidity. If the quantity of effused liquid continue to increase, the disease generally terminates fatally, from the excessive dyspnœa and the impediment to the action of the heart. If the disease pass into the chronic form, the condition of the liquid is changed, and becomes gradually purulent, as in other cases of serous inflammations.

The injection of the vessels of the pericardium is similar to that of other serous membranes. At first it is confined to a few dots and arborizations in the membrane, but gradually increases, until nearly the whole surface is of a bright arterial redness, covered with a fine vascular net-work. If these vessels be minutely examined, they will be found to be situated in that part of the serous membrane which adheres to the cellular tissue; the serous surface is still smooth and nearly transparent; in the same stage the lymph may be removed from the surface, and leave it transparent until adhesions begin to form; then the transparency and smoothness of the surface are gradually destroyed; and blood, and afterwards vessels, are formed in the lymph, and inosculate with those of the membrane. It is evident, therefore, that the question whether the serous membrane is really thickened or not, depends merely upon the application of the term membrane; if it be confined to the external layer, it is very certain that it is not rendered opaque until a late period of the disease; but there is an actual thickening of the internal or adherent layer.

The inflammation of the pericardium is not in general of a tuberculous nature; this complication is, however, occasionally met with, and the anatomical characters are then quite similar to those of tuberculous pleurisy.

Pericarditis, like other pectoral affections, is recognized in part by local and physical, and in part by general symptoms. The physical signs of pericarditis are quite as conclusive as those of any other pectoral affection, when well developed; but when the disease is slight, and attended with but little serous effusion, they are often insufficient for accurate diagnosis. These cases, however, constitute but a small proportion of the whole number. The signs depend upon the physical properties of the effused liquid, and the changes in the action of the heart proper. As the physical lesions are greatest in those cases in which the liquid is most abundant, the signs are then the most decided.

They are as follows: 1. *Signs of conformation.* The liquid, if in large quantity, will of course distend the walls of the chest, and give rise to a fulness in the præcordial region. This is rather pyramidal than oval in shape, and extends from the diaphragm, or base of the chest, to the third rib, if the quantity of liquid amount to a pint or more; laterally the fulness extends to the nipple, or a short distance beyond it. The rise is very gradual; hence it requires careful inspection, in many cases; to discover it.

2. *Percussion.* The evidence furnished by this mode of investigation is much more conclusive. If there be a decided prominence in the præcordial region, the percussion is of course flat to the same extent; for the prominence depends upon an effusion of liquid between the walls of the chest and the heart, thus forcing the lung aside, and destroying the healthy resonance of the chest. Percussion is, therefore, of immense value in those cases in which the effusion is large; but when there is little or no liquid, and the effusion consists almost entirely of lymph, there is comparatively little dulness, at least no more than would be caused by a heart moderately hypertrophied.

